# Mitral Annulus Calcification is associated with valvular and cardiac structural abnormalities

**DOI:** 10.1186/1476-7120-5-14

**Published:** 2007-03-14

**Authors:** Mohammad-Reza Movahed, Yuji Saito, Mastaneh Ahmadi-Kashani, Ramin Ebrahimi

**Affiliations:** 1 Department of Medicine, Section of Cardiology, University of Arizona Sarver Heart Center, Tucson, Arizona, USA; 2 Department of Medicine, Division of Cardiology, University of California, Irvine Medical Center, Irvine, California, USA; 3 Department of Medicine, Division of Cardiology, University of California, and the Greater Los Angeles VA Medical Center, Los Angeles, California, USA

## Abstract

**Introduction:**

Mitral annulus calcification (MAC) is a common finding on echocardiographic examination. The goal of this study was to evaluate associations between MAC and cardiac abnormalities using a large echocardiographic database.

**Methods:**

For this study we retrospectively reviewed 24,380 echocardiograms performed for clinical reasons between the years 1984 and 1998.

**Results:**

MAC was reported in 1,494 (6.1%) subjects. Using multivariate analysis, age, left ventricular hypertrophy (LVH), mitral regurgitation (MR), tricuspid regurgitation (TR), aortic stenosis (AS), left atrial (LA) enlargement and reversed E/A ratio were independently associated with MAC.)MAC was noted in 11.7 % of patients with MR vs. 4.3% without MR (OR: 2.0, CI 1.6–2.6, p < 0.0001), in 13.9% of those with TR vs. 4.5% without TR (OR: 3.8, CI 2.9–4.8, p < 0.0001), in 10.6% with LVH vs. 4.2% without LVH (OR: 1.9, CI 1.5–2.4, p < 0.0001), in 14.8% with AS vs. 5.5% without AS (OR: 1.4, CI 1.08–1.9, p = 0.01), in 9.4% with reversed E/A ratio vs. 3.8% without reversed E/A ratio (OR: 1.7, CI 1.4–2.2, p < 0.0001) and in 8.2% with LA enlargement vs. 4.8% without LA enlargement (OR: 1.3, CI 1.06–1.7, p = 0.02).

**Conclusion:**

In our study, MAC independently correlated with significant structural heart abnormalities. This suggests that identification of MAC may serve as a marker for other cardiac structural disorders.

## Background

**Figure 1:**
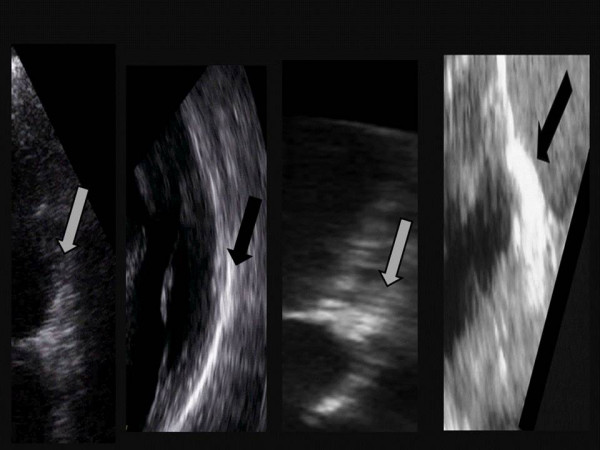
From left to right, examples of normal mitral annulus and calcified mitral annulus (mild = 1–2 mm, moderate = 2–5 mm and severe >5 mm calcified annulus).

Idiopathic (degenerative) mitral annular calcification (MAC) is one of the most common cardiac abnormalities found upon autopsy. Four examples of normal, mild, moderate and severe MAC can be seen in figure 1. Although the development of degenerative calcification of the mitral annulus is functionally of little consequence in most hearts, it shares common risk factors with atherosclerosis; including systemic hypertension, hypercholesterolemia, and diabetes [1]. Therefore it is important to risk-stratify patients with MAC because of its association with other important disorders such as coronary and carotid atherosclerosis and increased risk for cardiovascular morbidity and mortality [1-3].

It has also been suggested that MAC might become an important cause of mitral regurgitation (MR) when calcification is severe [4,5]. Previous observational studies suggest that MAC might be associated with several other cardiovascular disorders such as atherosclerosis, MR, stroke, atrioventricular conduction defects and hypertrophic cardiomyopathy [4-7]. In this study we analyzed a large echocardiographic database of over 24,000 patients. We performed comprehensive uni- and multivariate analyses to evaluate associations between MAC and cardiac abnormalities including valvular abnormalities, left ventricular hypertrophy (LVH) and left atrial (LA) enlargement.

## Methods

We retrospectively reviewed 24,380 echocardiograms performed between the years 1984 and 1998, which were ordered by clinicians for various indications. MAC and other echocardiographic findings were diagnosed based on the interpretation of echocardiograms by various cardiologists using echocardiographic parameters on a complete echocardiogram, including pulse and continuous wave Doppler, Doppler color flow imaging and spectral Doppler velocimetry studies. The documented final diagnoses were available and used for our study. The reports were generated by clinical cardiologists trained in the interpretation of echocardiograms. The diagnosis of regurgitations included mild to severe, but trace regurgitations that are commonly observed in healthy population were not included in the diagnosis of regurgitations. Using uni- and multivariate analysis, we evaluated the association between age, gender, valvular abnormalities, pericardial effusion, decreased fractional shortening (FS defined as FS < 25%), left ventricular hypertrophy (LVH defined as left ventricular wall thickness >11 mm measured in standard parasternal long axis view involving at least one of the ventricular walls), aortic thickening, aortic root enlargement (defined as aortic root over 35 mm measured in parasternal long axis), abnormal early over late mitral flow reversal (reversed E/A ratio), body mass index (BMI>25) and enlarged left atrial (LA defined as left atrial size > 40 mm measured in standard parasternal long axis view), tricuspid regurgitation (TR), aortic regurgitation (AR) and aortic stenosis (AS). The statistical analysis was performed using SPSS software. A p-value of less than 0.05 was interpreted as statistically significant. Variables were analyzed using χ^2 ^test. Quantitative variables were specified as mean ± standard deviation. In the multivariate analysis, a logistic regression model was utilized to calculate the odds ratio and their 95% confidence interval to indicate the strength of influence. We used SPSS statistical program version 13 for the statistical analysis. Baseline clinical characteristics of the patients were not available in the database. The institutional review board approved this study.

## Results

MAC was diagnosed in 1,494 (6.1%) of the 24,380 echocardiograms. Using univariate analysis, MAC was significantly associated with age, gender, MR, TR, AR AS, thickened aortic valve; abnormal early over late mitral flow reversal (reversed E/A ratio; dilated aortic root; LA enlargement; BMI >25 and LVH. The association between MAC and valvular regurgitations was independent of the severity of regurgitation. Using multivariate analysis, age, LVH, MR, TR, AS, LA enlargement and reversed E/A ratio were independently associated with MAC. Mean age of patients with MAC was 68 years vs. 50 years in those without MAC (OR: 1.059, CI 1.054–1.062, p < 0.0001).)MAC was noted in 11.7 % of patients with MR vs. 4.3% without MR (OR: 2.0, CI 1.6–2.6, p < 0.0001), in 13.9% of those with TR vs. 4.5% without TR (OR: 3.8, CI 2.9–4.8, p < 0.0001), in 10.6% with LVH vs. 4.2% without LVH (OR: 1.9, CI 1.5–2.4, p < 0.0001), in 14.8% with AS vs. 5.5% without AS (OR: 1.4, CI 1.08–1.9, p = 0.01), in 9.4% with reversed E/A ratio vs. 3.8% without reversed E/A ratio (OR: 1.7, CI 1.4–2.2, p < 0.0001) and in 8.2% with LA enlargement vs. 4.8% without LA enlargement (OR: 1.3, CI 1.06–1.7, p = 0.02). There was no association between MAC and mitral stenosis, decreased fractional shortening or pericardial effusion (Table 1).

**Table 1:** Multivariate Odds ratios (OR) and confidential intervals (CI) for the association between MAC and various cardiac abnormalities:

	**OR**	**95% CI**	***p***
LVH	1. 9	1.5–2.4	< 0.0001
MR	2.0	1.6–2.6	< 0.0001
TR	3.8	2.9–4.8	< 0.0001
AR	0.9	0.7–1.2	0.52
AS	1.4	1.08–1.9	0.01
LAE	1.3	1.06–1.7	0.02
E/A reversal	1.7	1.4–2.2	< 0.0001
Age > 50	4.8	3.5–.6.4	< 0.0001
Male gender	0.8	0.65–1.02	0.08
BMI >25	1.1	0.9–1.4	0.4
DAR	1.2	0.9–1.6	0.2

LVH = Left Ventricular Hypertrophy, MR = Mitral Regurgitation, TR = Tricuspid Regurgitation, AR = Aortic Regurgitation, AS = Aortic Stenosis, Age in years, LAE = Left Atrial Enlargement, BMI = Body Mass Index, DAR = Dilated Aortic Root.

## Discussion

Calcification within the mitral annulus results from a degenerative process in the cardiovascular fibrous skeleton, which is reported to be accelerated by advanced age, systemic hypertension, hypercholesterolemia, diabetes mellitus, chronic renal failure with secondary hyperparathyroidism and genetic abnormalities of the fibrous skeleton; such as, Marfan and Hurler syndromes [1,8,9]. The initial pathologic event is considered to be fibrillar alteration of the collagen ultrastructure [10]. This change triggers lipid deposition and the subsequent development of small foci of calcification deep within the annulus and at points of interdigitation between the annulus and ventricular muscle fibers.

Our study was conducted in order to evaluate association between MAC and age, gender and other echocardiographic cardiac abnormalities using a large number of echocardiograms. We demonstrated that MAC was significantly associated with advanced age, valvular regurgitations such as MR, TR and aortic stenosis, but not AR or MS. It is suggested that when the mitral valve annulus becomes thick, rigid and calcified, it may interfere with valve closure, causing MR. In this setting, age is considered to play a significant role in mitral valve degeneration, triggering MAC. This association also explains the association between advanced age and the occurrence of MAC. The reason for MAC association with TR is not known. A secondary increase in the pulmonary arterial pressure due to associated MR or AS may explain this association. Furthermore, patients with MAC are also at risk for calcification in other vascular systems as a marker of atherosclerosis and aging [3,6,8,11,12]. However, the independent association of MAC with AS in our study suggests that MAC may play a direct role in the pathogenesis of AS or that patients with MAC may represent a group of individuals with increased susceptibility to generalized calcium deposition in the cardiac and extra cardiac tissue. Fulkerson et al., on the other hand, reported that conditions associated with chronically increased left ventricular pressures, such as aortic stenosis and systemic hypertension, enhance stress on the mitral valve and apparatus or promote abnormal valve motion. These effects are considered to accelerate the degenerative process, leading to premature calcium deposition as a possible explanation of our findings [10]. Furthermore, our large population study confirms Fulkerson et al.'s observation that MAC was significantly associated with LVH and AS. Coronary artery disease and systemic calcified atherosclerosis have been found to be strongly associated with MAC suggesting similar pathogenesis in calcium deposition involving cardiovascular system [13,14]. Our study failed to show any significant association between MAC and AR. It appears that MAC does not decrease the integrity of aortic valve that could lead to AR. We have a small number of patients with mitral stenosis in our database limiting the assessment of the association of MAC and mitral stenosis. Finaly, MAC has been found to be associated with significant cardiovascular morbidity and mortality [15] confirming our finding that the presence of MAC could be marker for underlying cardiovascular pathology.

## Conclusion

This is the first large-scale study demonstrating that MAC is independently associated with advanced age, MR, TR, AS, diastolic mitral flow reversal, left atrial enlargement and LVH. This suggests that identification of MAC may serve as a marker for other cardiac structural disorders and that patients with MAC may require further evaluation.

## Limitations

This is a retrospective study that relied on previous reports, resulting in non-uniform assessment of the echocardiographic abnormalities. The diagnoses of cardiac abnormalities were not standardized. Data regarding criteria for diagnosis and grading of MAC was not available. The sensitivity of the older equipments for detection of MAC is probably less than the newer equipments. We scored MAC in our study as absent or present. However, a semiquantitative assessment of MAC severity could have improved the study interpretation.
